# Effects of a 12-Week Aerobic Spin Intervention on Resting State Networks in Previously Sedentary Older Adults

**DOI:** 10.3389/fpsyg.2018.02376

**Published:** 2018-11-27

**Authors:** Keith M. McGregor, Bruce Crosson, Lisa C. Krishnamurthy, Venkatagiri Krishnamurthy, Kyle Hortman, Kaundinya Gopinath, Kevin M. Mammino, Javier Omar, Joe R. Nocera

**Affiliations:** ^1^VA Rehabilitation R&D Center for Visual and Neurocognitive Rehabilitation, Atlanta VA Health Care, Decatur, GA, United States; ^2^Department of Neurology, School of Medicine, Emory University, Atlanta, GA, United States; ^3^Department of Psychology, Georgia State University, Atlanta, GA, United States; ^4^Department of Physics & Astronomy, Georgia State University, Atlanta, GA, United States; ^5^Department of Radiology, School of Medicine, Emory University, Atlanta, GA, United States; ^6^Division of Physical Therapy, School of Medicine, Emory University, Atlanta, GA, United States

**Keywords:** aging, resting state, motor control, functional connectivity, aerobic exercise

## Abstract

**Objective:** We have previously demonstrated that aerobic exercise improves upper extremity motor function concurrent with changes in motor cortical activity using task-based functional magnetic resonance imaging (fMRI). However, it is currently unknown how a 12-week aerobic exercise intervention affects resting-state functional connectivity (rsFC) in motor networks. Previous work has shown that over a 6-month or 1-year exercise intervention, older individuals show increased resting state connectivity of the default mode network and the sensorimotor network (Voss et al., 2010b; Flodin et al., 2017). However, the effects of shorter-term 12-week exercise interventions on functional connectivity have received less attention.

**Method:** Thirty-seven sedentary right-handed older adults were randomized to either a 12-week aerobic, spin cycling exercise group or a 12-week balance-toning exercise group. Resting state functional magnetic resonance images were acquired in sessions PRE/POST interventions. We applied seed-based correlation analysis to left and right primary motor cortices (L-M1 and R-M1) and anterior default mode network (aDMN) to test changes in rsFC between groups after the intervention. In addition, we performed a regression analysis predicting connectivity changes PRE/POST intervention across all participants as a function of time spent in aerobic training zone regardless of group assignment.

**Results:** Seeding from L-M1, we found that participants in the cycling group had a greater PRE/POST change in rsFC in aDMN as compared to the balance group. When accounting for time in aerobic HR zone, we found increased heart rate workload was positively associated with increased change of rsFC between motor networks and aDMN. Interestingly, L-M1 to aDMN connectivity changes were also related to motor behavior changes in both groups. Respective of M1 laterality, comparisons of all participants from PRE to POST showed a reduction in the extent of bilateral M1 connectivity after the interventions with increased connectivity in dominant M1.

**Conclusion:** A 12-week physical activity intervention can change rsFC between primary motor regions and default mode network areas, which may be associated with improved motor performance. The decrease in connectivity between L-M1 and R-M1 post-intervention may represent a functional consolidation to the dominant M1.

**Topic Areas:** Neuroimaging, Aging.

## Introduction

Aerobic exercise is an inexpensive, ecological lifestyle intervention that has long been shown to benefit movement and cognition in older adults (Larson and Bruce, 1987; Hatziandreu et al., 1988). Previous work has shown that aerobic exercise can increase overall brain volume (Colcombe et al., 2006), increase hemispheric gray matter volume (Erickson et al., 2014), increase hippocampal size (Erickson et al., 2011; Szabo et al., 2011), and alter patterns of task-based cortical activity (Erickson, 2007; McGregor et al., 2011; Zlatar et al., 2013). While the beneficial impact of aerobic exercise on brain density and cortical activity is becoming increasingly established, much less is known about the effect of exercise intervention on resting state connectivity particularly in older adults. Because functional connectivity is a fundamental aspect of neuronal communication required for high-level cognitive processes, it is important to understand the potential impact of aerobic exercise.

A seminal study by Voss et al. (2010b) first showed significant increases in resting state functional Magnetic Resonance Imaging (rsfMRI) connectivity in the default mode network (DMN) and frontal executive networks in older adults after a 1 year aerobic exercise intervention (Voss et al., 2010b). Similarly, a cross-sectional study of resting state networks in older adults showed that older adults who engaged in higher levels of physical activity over 10 years (the Betula Study - Umea University, Sweden) showed increased DMN connectivity, particularly in posterior cingulate regions (Boraxbekk et al., 2016). A recent study by (Flodin et al., 2017) investigated changes in resting state connectivity across different known task networks. The study employed a randomized controlled design whereby 60 participants were randomized into either an aerobic exercise condition (walking, cycling, stair-stepping) or a toning control group involving repetitive muscle toning movements (air squats, light weights, isometric contraction, etc.). The authors reported that both groups significantly increased VO_2_ peak over the 6-month interventions (thrice weekly sessions). In addition, no differences between groups were evident in comparisons of resting state network connectivity changes PRE to POST (Flodin et al., 2017). Interestingly, when the authors tested the change in VO_2_ peak as a predictor of changes in functional network connectivity, they reported that increased VO_2_ peak correlated with differential connectivity of two resting-state networks: the DMN and the sensorimotor network (M1S1).

While previous work has shown exercise-induced connectivity changes within the frontal executive, default mode, and M1S1 networks (Burzynska et al., 2015; Flodin et al., 2017), the connectivity between these networks has received less attention. Few studies have compared seedings from regions of the motor network to investigate changes in the DMN, and vice versa. Previous work has shown that increased physical activity in older adults may modify patterns of task-based fMRI activity within the primary sensorimotor cortices that are associated with improvements in motor function (Voelcker-Rehage et al., 2010; McGregor et al., 2011, 2013) and increases in transcallosal inhibition as measured by transcranial magnetic stimulation (McGregor et al., 2018). Improved (or retained) connectivity within the DMN in aging is believed to be associated with improved executive control, particularly in anterior prefrontal cortices (Vidal-Pineiro et al., 2014; Siman-Tov et al., 2017).

While aerobic capacity has been postulated as the dominant agent of change in physical activity interventions (Voss et al., 2016), there is evidence that any physical activity intervention may induce changes in cortical connectivity (Boraxbekk et al., 2016; Flodin et al., 2017; Jonasson et al., 2017; Prehn et al., 2017). This may be due to the sedentary nature of the samples entering such interventions. As such, instantaneous measures of physical activity (estimated VO_2_max/peak) in this population may not be the best metric to index changes in physiology across the intervention. For example, in Burzynska et al. (2015), when comparing cardiorespiratory fitness (CRF) (measured by a VO_2_max test) and physical activity (as measured by 7-day accelerometry), the authors reported that differences in motor network and DMN were best described by amount of physical activity rather than single instance measures of CRF (Burzynska et al., 2015).

In the present study, we enrolled 56 previously sedentary older adults (60+ years) and randomized them into either a 12-week aerobic based interval spin program or a stretching and balance control group matched for duration and contact with study personnel. We obtained resting state functional magnetic resonance imaging at baseline (PRE) and after the 12-week interventions (POST). We report on changes in functional connectivity after the physical activity interventions in seeds from the primary motor cortex and default mode network. Our central hypothesis is that 12-weeks of aerobic training changes patterns of activity in resting state motor networks with increased connectivity within motor networks. We further hypothesize that resting state networks associated with internal control (i.e., DMN and executive networks) will exhibit a higher level of connectivity after aerobic interventions indicating improved top-down regulation of motor control.

## Materials and Methods

### Participants

**FIGURE 1:**
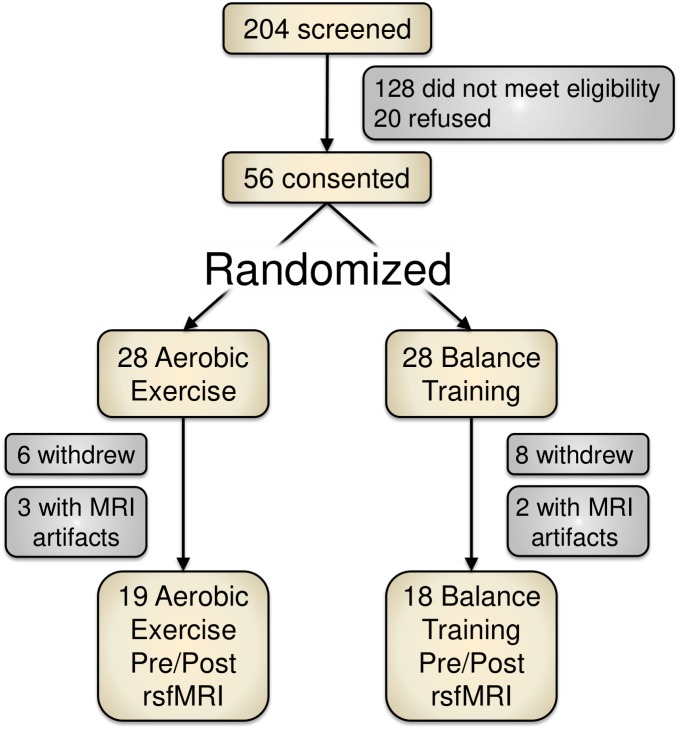
Study recruitment and participant flowchart. Of the 204 screened participants, 56 provided informed consent. Due to attrition or imaging artifacts, we report PRE/POST data from 37 participants (19 aerobic exercise; 18 balance training).

Of 204 screened individuals, 56 older adults (ages 60–80) provided informed consent for participation. These participants were randomized into either an aerobic exercise training condition (spin cycling) or a balance and stretching exercise training group. Figure 1 depicts a recruitment and participation flowchart. Due to study attrition (*n* = 14) or poor MRI data (*n* = 5), we removed 19 participants from data analysis for this report.

We report resting-state functional MRI data on 37 sedentary right-handed older adults (60 years or older) prior to and after participating in a 12-week physical activity intervention. In this study, participants were randomized and divided into an aerobic, spin cycling exercise group (AE) or a non-aerobic balance training group (BAL) to equalize contact and monitoring. Study enrollment flowchart is depicted in Figure 1.

These data were acquired from two separate but overlapping projects using the same intervention. Study personnel explained the purpose, potential risks of the experiment and completed the informed consent process with each participant following protocols approved by the Emory University’s Institutional Review Board (IRB00059193, and IRB00056726) and Atlanta VA Research and Development Office. This study was carried out in accordance with the recommendations of Emory University’s Institutional Review Board and with oversight from the Atlanta VA Research and Development office with written informed consent from all subjects. All subjects gave written informed consent in accordance with the Declaration of Helsinki. The protocols were approved and overseen by both the Emory Institutional Review Board and the Atlanta VA Research and Development committee.

To meet inclusion criteria participants had to: (1) be between of 60–85 years of age, (2) report being sedentary, defined as not engaging in structured physical activity and/or not accumulating 30 min or more of moderate to strenuous weekly physical activity, assessed with a modified Godin Leisure Time Exercise Questionnaire – LTEQ (Godin and Shephard, 1985), (3) have no history of major psychiatric or neurological disease, (4) report being right handed (confirmed with a modified Edinburgh handedness inventory), (5) report being a native English speaker, and (6) obtain physician’s approval for study participation. Exclusion criteria included: (1) conditions that would contraindicate MRI, (2) failure to provide informed consent, (3) hospitalization within the past 6 months, (4) uncontrolled hypertension or diabetes, (5) inability to walk 400 m and (6) significant cognitive executive impairment, defined as a score on the Montreal Cognitive Assessment (MoCA) of <24 and poor performance (<30) on the American National Adult Reading Test (NART).

During all intervention sessions, participants wore a Polar (Bethpage, NY, United States) FT7 chest strap heart rate monitor with paired monitor/wristwatch. Heart rate was taken from each participant every 2–3 min during the sessions and logged on a data sheet. On infrequent occasions (<2% of HR acquisitions), the chest strap monitor would fail to synchronize with the watch during the intervention session. If a heart rate monitor failed to synchronize at study outset (a problem with older adults with lower resting galvanic skin responses) we would use a battery-powered pulse oximeter or an Apple Watch (Cupertino, CA, United States) to measure heart rate at the above described intervals. For both interventions, we recorded attendance, attrition, and heart rate.

### Aerobic ‘Spin’ Intervention Protocol

Consistent with our previous exercise studies (Nocera et al., 2017; McGregor et al., 2018), the AE and BAL interventions were both three times weekly for 12-weeks and commenced with 20-min sessions. The time of each session was equalized across groups and progressed by 1–2 min/session as needed to a maximum time of 45 min per session. Both interventions were group based and led by qualified instructors (ACSM Certified).

The Spin intervention was an interval-based program (5-min warm-up, then steady up-tempo cadences, sprints, and climbs, followed by a 5-min cool down) in which overall intensity began at 50% target intensity and increased by 5% every week (if deemed necessary by the instructor) to a maximum of 75% target intensity. Target HR was computed using the Karvonen method shown in Equation 1:

(1) Target HR=((220−age)−resting heart rate)•(%target intensity)+resting heart rate

During the workout phase the Target HR was maintained by averaging increases and decreases in HR. The goal was to maintain a 10% offset from the Target HR goal during the workout phase. Thus, the program was designed to have an average workload within Target HR across the session despite the intervals of increased and decreased workload. Staff members monitored and tracked the HR to encourage adequate intensity throughout each session.

### Balance/Strength Training Intervention Protocol

Participants in the balance group were equalized to the Spin group with regards to contact and monitoring frequency. This intervention arm took place in the same facility with the same study personnel; however, instead of progressive aerobic exercise they participated in group balance, stretching and light muscle toning exercises (5-min stretching, 10-min of balance exercises, and light strength training). Heart rate was consistently monitored (also using the Polar FT7 chest strap monitors) to assess general intensity during each session and to advise participant to keep HR below aerobic levels (defined as below a Target_50_ HR, where

$${\mathrm{Target}}_{50}\;\mathrm{HR}=((220-\mathrm{age})-\mathrm{resting}\;\mathrm{heart}\;\mathrm{rate})•(50\%)+\mathrm{resting}\;\mathrm{heart}\;\mathrm{rate)}.$$

### Cardiovascular Fitness Assessment

To assess aerobic capacity, participants performed a YMCA submaximal fitness test on a Monark 828e (upright) or RC4 (recumbent) cycle ergometer (Vansbro, Sweden) to estimate the participant’s maximal oxygen uptake (VO_2_max) prior to and after interventions. The YMCA test uses an extrapolation method in which heart rate workload values are obtained at 2–4 points during stages of increasing resistance and extrapolated to predict workload at the estimated maximum heart rate (e.g., 220-age). VO_2_max is then calculated from the predicted maximum workload. Prior to beginning the test, the participants completed a 2-min warm-up consisting of pedaling without load so that they could adapt to the ergometer for the first minute and then pedaling with a 0.5 kg.m load during the second minute. As progressive load is applied to the braking of the cycle, some participants (*n* = 9) failed to reach the 9-min mark during the test, in which case the first two data points are used to project the VO_2_max.

### Heart Rate Workload Assessment

In the present study, our participants were highly sedentary. As such, the projected assessment of VO_2_max using the YMCA test was more variable than expected as some participants had difficulty reaching target cycle resistance for an accurate measure. In addition, many participants in our balance training condition had sustained heart rates at or above aerobic training levels (above Target_50_ HR). While this is a noted limitation in the study design, the higher-than-expected heart rate workloads in the balance training groups offered an opportunity to collapse participants across groups respective of heart rate workload, as both groups had participants that consistently engaged in aerobic training.

To better capture the actual HR performance of our participants during training sessions, we identified the percentage of time our participants were at or above Target_50_ HR across exercise sessions. In each session, all participants’ heart rates (regardless of group assignment) were obtained every 2–3 min. These data were transcribed and denoted to be either at or above Target_50_ HR (i.e., light aerobic zone). Then we calculated the percentage of total intervention heart rate assessments at or above target heart rate (about 320 assessments per participant), which we will call % Time in Target_50_ HR Zone as shown in Equation 2:

(2) $$\%\mathrm{Time}\;\mathrm{in}\;{\mathrm{Target}}_{50}\;\mathrm{HR}\;\mathrm{Zone}=\frac{\#\;\mathrm{HR}\;\mathrm{acquisitions}\;\geq \;{\mathrm{Target}}_{50}\;\mathrm{HR}}{\mathrm{total}\;\#\;\mathrm{HR}\;\mathrm{acquisitions}}$$

### Motor Testing

Participants in the current study performed a battery of dexterity and psychomotor speed measures that included the: Halstead-Reitan Tapping Assessment, the 9-hole pegboard test, the Purdue pegboard test, and grip strength assessments. The Halstead-Reitan tapping test is a test of putative psychomotor processing speed that involves repetitively pressing down a small lever as quickly as possible within a ten-second interval. Participants are asked to perform the test across up to ten trials (ten 10-s intervals) or to stop if five consecutive trials are within five presses of each other (e.g., 30,34,32,33,32). Performance is averaged across the trials. The 9-hole peg test requires each participant to place pegs in a field of 9 holes and then remove the pegs as quickly as possible (performance is measured in time to complete). The Purdue pegboard task has two components. The first component requires participants to fill a field of peg holes with cylindrical pegs in sequence (performance is measured in how many pegs are placed within 30 s). The second component of Purdue is an assembly task during which a peg is covered by a washer and then capped with a collar in serial fashion (performance is measured in the total number of 3-part assemblies in 1 min). Grip strength is measured by isometric grip squeeze at maximal force as measured by a Jamar-brand hand dynamometer.

### MRI Acquisition

The MRI scans were acquired on either a Siemens 3T Tim Trio MRI scanner or a Siemens 3T Prisma-FiT MRI (Erlangen, Germany) using the body coil for radio frequency (RF) transmission and a 12-channel phased-array head coil for RF receiving. The PRE and POST scans for each subject were completed on the same MRI platform (either the Trio or Prisma) to minimize the influence from scanner differences. A high-resolution T1-weighted 3D magnetization prepared rapid acquisition gradient echo (MP-RAGE) scan (TE = 3.02 ms, TR = 2600 ms; FOV = 240 mm; FA = 8°; matrix size = 256 × 256, 176 × 1.0 mm sagittal slices) was obtained to provide anatomic reference. Head motion was minimized using foam padding and careful instructions were given to the participant to avoid moving. During the resting state scan, the subjects were instructed to gaze at a white fixation cross on a black background. The rsfMRI time course was acquired with a single shot gradient recalled echo planar imaging (EPI) sequence (FoV = 220 mm × 220 mm, matrix = 74 × 74, 48 slices, slice thickness = 3 mm, TR = 3000 ms, TE = 24 ms, FA = 90°, 192 measurements, acquisition time = 9:36 min). Of note, due to study MRI protocol differences, resting state acquisition was acquired immediately after MPRAGE acquisition in 20 participants (cohort 1), but in 17 participants (cohort 2), additional scans (diffusion weighted and task fMRI) were acquired between MPRAGE and resting state. For data integrity and possible influence of the additional scans in cohort 2, we performed a between-subjects *t*-test comparison across study cohorts and found no differences in resting state activity after data preprocessing. As such, data were collapsed across cohorts.

### MRI Pre-processing

The MR images were processed using AFNI, FSL, and ITKSnap software packages, and in-house Matlab scripts. The rsFC MRI time course was corrected for slice-timing and global head motion. Then systematic hardware, physiological, and motion related artifacts were removed with Independents Component Analysis (ICA) using study-specific, hand-trained classifiers created from a subset of the rsfMRI sample (12 datasets; 6 pre and 6 post) via FSL’s Melodic and FIX. Spatial normalization to MNI template space was performed in conjunction with the MPRAGE using non-linear transforms. Frame-to-frame displacement was computed (Power et al., 2014) to censor the rsfMRI time series at a 0.5 mm threshold. Data from 5 participants had excessive (>40% of images beyond censor threshold) movement artifacts and were discarded allowing for us to include 37 of the 42 participants who completed the protocol in analyses. The ventricles were masked in the rsfMRI time series to reduce the influence from cerebrospinal fluid pulsatility. A low-pass filter was applied to the rsfMRI time series using a Chebyshev II filter with cut-off frequency of 0.1 Hz (Krishnamurthy et al., 2015), and smoothed with a 5 mm full-width-half-maximum Gaussian filter.

### Seed-Based rsfMRI Analysis

We chose the seeds based on *a priori* knowledge of left hemisphere primary motor cortex (Yousry et al., 1997) situated on the L-M1 hand-bump (MNI coordinates *x* = 35.7, *y* = 26.9, *z* = 51.8), analogous right hemisphere R-M1 hand-bump (*x* = -33.3, *y* = 23.4, *z* = 53.4), and anterior node of the default mode network (aDMN; *x* = 0, *y* = -47.4, *z* = 16.2). A 5 mm radius sphere at the seed MNI coordinates (Gopinath et al., 2015) was used to extract an average seed time course to cross-correlate with the time courses of all other voxels. The Fisher z-transform was applied to the cross-correlation values to normalize the distribution, and is denoted as Z(CC).

### Evaluating Group Differences Between AE and BAL

To determine the effects of the aerobic spin exercise program compared to the balance program, the difference in Z(CC) between PRE and POST scans were evaluated on a voxel-wise basis for each subject as shown in Equation 3.

(3) $$Z{\left(CC\right)}_{\mathrm{DIFF}}=Z{\left(CC\right)}_{\mathrm{POST}}-Z{\left(CC\right)}_{\mathrm{PRE}}$$

The assumption here is that the Z(CC)_DIFF_ removes the within-subject variability to improve the detectability of brain changes due to the exercise intervention. Then the Z(CC)_DIFF_ was compared across groups via *t*-test (*p* = 0.01, cluster = 50, FWE corrected) for every seed.

### Evaluating Relationship Between % Time in Target_50_ HR Zone and Z(CC)_DIFF_

Using in-house Matlab scripts, the linear relationship between % Time in Target_50_ HR Zone and Z(CC)_DIFF_ were evaluated to determine if the level of exercise intensity was related to the amount of changes in connectivity. Using a Levenberg-Marquardt non-linear least squares algorithm (Matlab’s nlinfit), the coefficients A (intercept) and B (slope) in Equation 4 were estimated based on the % Time in Target_50_ HR Zone and Z(CC)_DIFF_ data at a voxel-wise basis.

(4) $$\%\mathrm{Time}\;\mathrm{in}\;{\mathrm{Target}}_{50}\mathrm{HR}\;\mathrm{Zone}=A+B•Z{\left(\mathrm{CC}\right)}_{\mathrm{DIFF}}$$

The significance of the relationships was determined via the *R*^2^ metric (e.g., how much variance in Z(CC)_DIFF_ is accounted for by % Time in Target_50_ HR Zone).

### Evaluating Relationship Between Changes in Motor Behavior and Z(CC)_DIFF_

A subset of 20 participants (cohort 1) performed a battery of cognitive and upper extremity motor tests PRE and POST 12-week exercise intervention as described in McGregor et al. (2018), including the Halstead-Reitan Finger Tapping task (Reitan and Wolfson, 1986). We selected the Halstead-Reitan battery due to its sensitivity to reported aging-related changes in psychomotor speed (Elias et al., 1996). To determine if exercise induced changes in functional connectivity predicted changes in Halstead, brain-behavior relationships were calculated with a linear regression analysis on a voxel-wise basis as described in Equation 5:

(5) $${\mathrm{Halstead}}_{\mathrm{POST}-\mathrm{PRE}}=A+B•Z{\left(\mathrm{CC}\right)}_{\mathrm{DIFF}}$$

The resulting brain-behavior regression maps were thresholded at *p* = 0.01 and clusterized at 50 voxels.

### Evaluating Hemispheric Connectivity Differences in M1

The rsfMRI connectivity profile was computed separately for left M1 [Z(CC)_Left_M1] and right M1 [Z(CC)_Right_M1] in PRE and POST sessions for each subject. Then the hemispheric asymmetry (L-R_M1_) in connectivity was assessed based on Equation 6:

(6) $$L-R_{\mathrm{M1}}=Z{\left(\mathrm{CC}\right)}_{\mathrm{Left}\;M1}-Z{\left(\mathrm{CC}\right)}_{\mathrm{Right}\;M1}$$

The L-R_M1_ value is positive if L-M1 is more connected to a voxel than R-M1, and negative if R-M1 is more connected to a voxel than L-M1. The L-R_M1_ metric is close to zero if the left and right M1 have a similar connection strength to a voxel. Significant areas of hemispheric connectivity differences are identified in PRE and POST via *t*-test compared to 0 (*p* = 0.01, cluster = 50, FWE corrected) by collapsing across groups. The resulting clusters on the right and left hemispheres are identified, and expressed as a percentage of the total number of significant voxels.

## Results

### Participant Demographics

Thirty-seven participants that completed the 12-week AE or BAL intervention (Age = 73.5 ± 6.8 years, Education = 14.6 ± 3.1 years, 23 Female) had usable PRE and POST MRI scans. Tables 1, 2 show demographics in each group. No significant differences were shown between groups due to randomization.

**Table 1:** Baseline (PRE) demographics between groups.

Metric	Aerobic exercise (*N* = 18, 12 female)	Balance (*N* = 19, 12 female)
BMI	29.5 (3.5)	31.8 (4.7)
Estimated VO_2_ (ml/min/kg)	16.6 (7.4)	18.2 (6.5)
Godin LTEQ	27.2 (12.1)	24.1 (8.2)
MoCA	26.8 (1.6)	26 (2.9)


*No significant differences. The parenthetical numbers represent standard deviation*.

**Table 2:** Post demographics between groups.

Metric	Aerobic exercise (*N* = 18, 12 female)	Balance (*N* = 19, 12 female)
BMI	28.4 (7.8)	31.2 (8.6)
Estimated VO_2_ (ml/min/kg)^∗^	23.7 (6.2)^∗^	17.1 (4.2)^∗^
Godin LTEQ	24.1 (22.1)	13.3 (13.4)


*^∗^ - denotes p < 0.05 difference between groups. The parenthetical numbers represent standard deviation*.

After the interventions, participants in the AE group had significantly higher estimated VO_2_ as compared to the BAL group. Our screening measure of physical activity (Godin LTEQ) showed a significantly positive relationship with estimated VO_2_ max, *r*_s_ = 0.48, *p* = 0.03 in POST measurements.

### Intervention Effects – Spin vs. Balance

**FIGURE 2:**
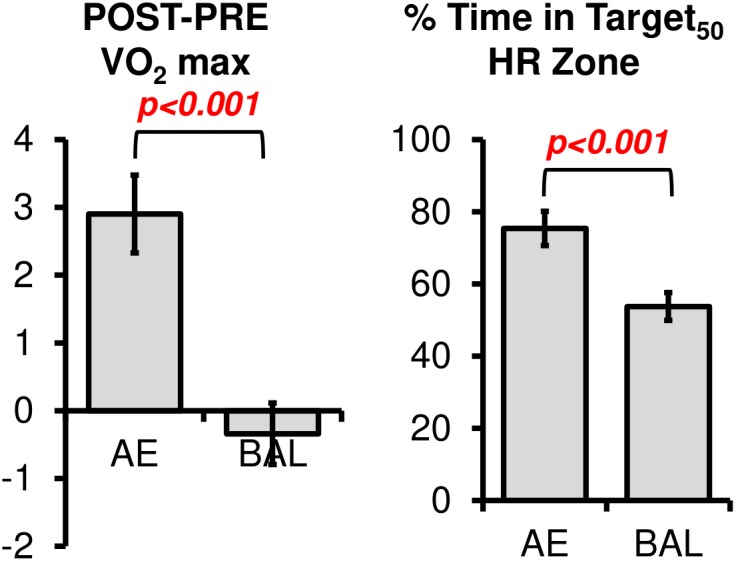
The changes in VO_2_ max were significantly different between AE and BAL groups, indicating the success of the aerobic spin intervention. The AE group also spent a greater amount of time in the prescribed Target HR Zone during their 12 weeks intervention, as indicated by the % Time in HR Target Zone.

Comparisons between groups on the YMCA submaximal cycling test revealed significant post intervention change in VO_2_max between types of intervention [Figure 2, *t*(36) = 3.76, *p* < 0.001]. However, we noted outliers within this analysis particularly at PRE. That is, some participants had difficulty obtaining reliable steady state HR measures at the predetermined workload in the third and most important stage in the YMCA assessment. We did not see significant gender effects for VO_2_ change.

The % Time in Target_50_ HR Zone was computed according to Equation 2. As expected, % Time in Target_50_ HR Zone was higher for AE (mean = 75.3, *sd* = 16.8) as compared to BAL (mean = 53.7, *sd* = 20.1), [Figure 2, *t*(36) = 3.53, *p* < 0.001]. Of note, the % Time in Target_50_ HR Zone for the Balance intervention was higher than intended in the study design. We tested if heart rate workload was associated with gender in the current sample, as previous literature has related changes in neurotrophic factors (such as brain derived neurotrophic factor, a potential mechanism for changes in cortical plasticity) to heart rate workload within specific gender cohorts (Rojas Vega et al., 2006; Winter et al., 2007; Goekint et al., 2010). No gender effects were evident for heart rate data, *t*(35) = 0.46, ns.

### Seed-Based rsfMRI Analysis

**FIGURE 3:**
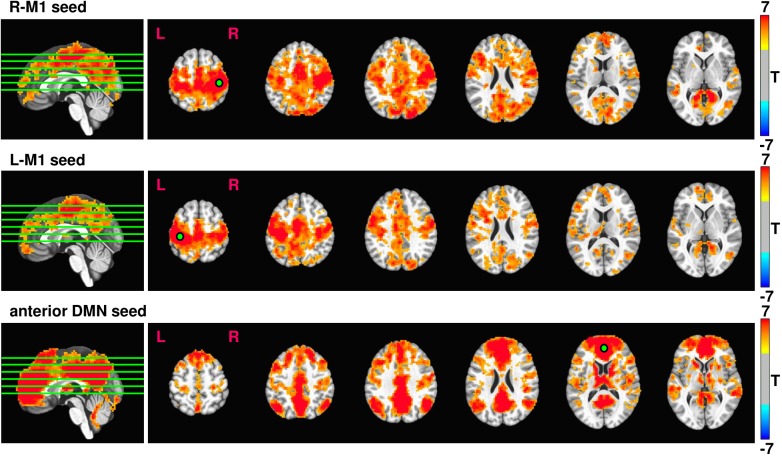
Resulting rsfMRI connectivity maps from R-M1, L-M1, and aDMN seeds. The intensity of the connectivity maps represent T score, thresholded at p = 0.0001, cluster size = 100. The green lines overlaid on the sagittal image represent the location of the displayed axial slices.

Average rsfMRI connectivity maps across all 37 subjects prior to 12 weeks exercise intervention seeded in R-M1, L-M1, and aDMN are shown in Figure 3. Expected connectivity profiles were found for every subject and across groups. When seeding from either left or right M1, the supplementary motor areas (SMA), dorsal and ventral premotor cortex (PMd and PMv), cingulate motor areas (CMA), and primary sensory areas (S1) showed significant connectivity. When seeding from aDMN, the posterior DMN, and left and right inferior parietal lobule (IPL) were strongly connected.

### Effects of 12 Weeks Exercise Intervention on Brain Connectivity

**FIGURE 4:**
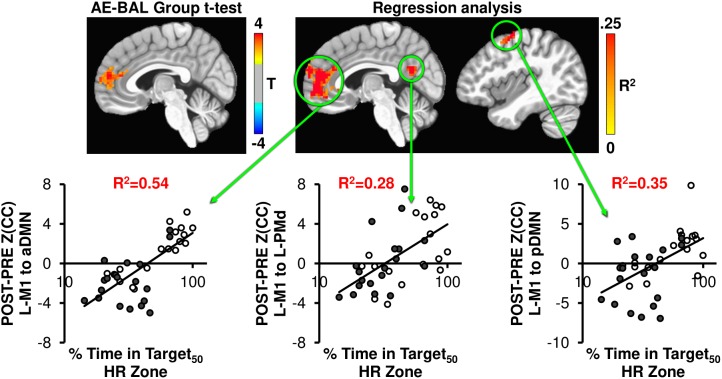
The AE-BAL group *t*-test of Z(CC)_DIFF_ seeded from L-M1 shows aDMN connectivity differences, indicating that the 12-week aerobic spin intervention was able to significantly L-M1 to aDMN connectivity in the AE group (*p* = 0.01, cluster size = 50). To further understand the relationship between individual subject response to the intervention, the % Time in Target HR Zone was correlated with Z(CC)_DIFF_ seeded from L-M1 on a voxel-wise basis as described in Equation 4. The regression analysis also shows exercise induced changes in L-M1 to aDMN connectivity, along with L-M1 to pDMN, and L-M1 to L-PMd connectivity (*p* = 0.01, cluster size = 50). The plots show the average Z(CC)_DIFF_ from the extracted ROI is predicted significantly by % Time in Target HR Zone. Closed circles = AE; Open circles = BAL.

**FIGURE 5:**
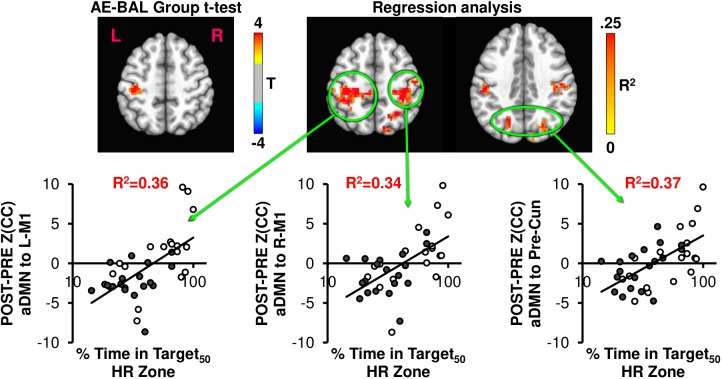
The AE-BAL group *t*-test of Z(CC)_DIFF_ seeded from aDMN shows L-M1 connectivity differences, which is expected based on the L-M1 seed results. However, when taking into account the individual subject response to the intervention by regressing Z(CC)_DIFF_ with % Time in Target HR Zone, both aDMN to L-M1 and aDMN to R-M1 connectivity relationships became apparent. A bilateral aDMN to Precuneus connectivity relationship also emerged with the regression analysis. These results indicate the importance of taking into account the individual subject response to the intervention to ascertain exercise-induced brain changes.

We hypothesized that our 12-week exercise intervention would show detectable changes in brain connectivity of the motor system, which other groups have shown in longer exercise interventions (Burdette et al., 2010; Voss et al., 2010a; Flodin et al., 2017). To probe this hypothesis, the Z(CC)_DIFF_ images were compared across the AE and BAL group via *t*-test, and also regressed with % Time in Target_50_ HR Zone for both L-M1 and aDMN seeds. Both seed regions showed unique brain connectivity increases with 12 weeks exercise intervention (Figures 4, 5).

As seen in Figure 4, the L-M1 to aDMN connectivity increases with AE compared to BAL. Interestingly, this increase in connectivity is more apparent when regressed with the % Time in Target_50_ HR Zone, as the regression analysis does not make assumptions on group differences in exercise intensity. This is important, as some of the AE participants (open circles in regression plots in Figures 4, 5) did not spend a great amount of time above their Target HR, whereas some of the BAL participants did (filled circles in regression plots in Figures 4, 5). This could cause a diminished separation in physiological input across groups during the 12-week intervention, which could affect the detectability of group differences in brain connectivity changes. The strongest relationship with % Time in Target_50_ HR Zone was between L-M1 and aDMN (*R*^2^= 0.54), which is also the region of detectable group differences using *t*-test comparisons (Figure 4). Interestingly, change in estimated VO2 (as assessed by the YMCA submaximal test) did not show significant correlations with frontal lobe connectivity.

Figure 5 shows the connectivity changes when seeded from aDMN, with the expected aDMN and L-M1 group differences. Regression analysis between aDMN Z(CC)_DIFF_ and % Time in Target_50_ HR Zone shows additional regions of significant exercise-induced brain connectivity increases, including L-M1, R-M1, and precuneus. Thus, the regression analysis indicates that the 12-week exercise intervention has a bilateral action in changing connectivity, which was not detectable by making assumptions on physiological group differences.

As shown in Figures 4, 5, across interventions, the greatest amount of variability is explained not by group assignment in the present study, but rather by heart rate activity across the sessions. As such, the subsequent analyses report on total intervention effect rather than within or between group comparisons.

### Predicting Changes in Motor Behavior With Brain Connectivity Changes

**FIGURE 6:**
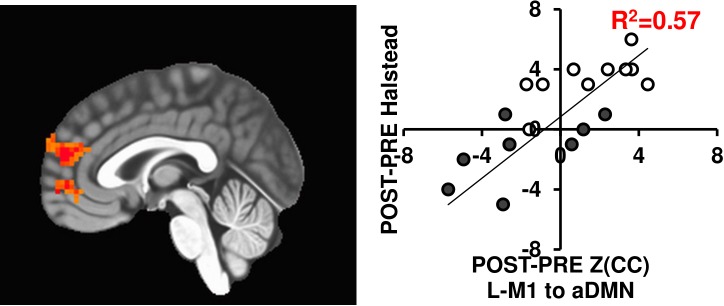
The L-M1 to aDMN Z(CC)_DIFF_ significantly predicts the amount of change in the Halstead test of Psychomotor speed (*p* = 0.01, cluster size = 50). The extracted ROI Z(CC)_DIFF_ accounts for 57% of variance in the change in Halstead score after a 12-week intervention. Closed circles = AE; Open circles = BAL.

Since brain connectivity changes themselves do not necessarily translate to functional outcomes, we tested if the connectivity changes related with motor behavior changes using the Halstead measure of psychomotor speed. As seen in Figure 6, the L-M1 to aDMN connectivity increases positively relate with changes in Halstead PRE/POST in either 12-week exercise intervention (*R*^2^= 0.57). While this is in a subset of individuals (*N* = 20), previous work has shown improved motor performance based across sessions in similar interventions (see McGregor et al., 2018).

### Hemispheric Connectivity Differences in M1

**FIGURE 7:**
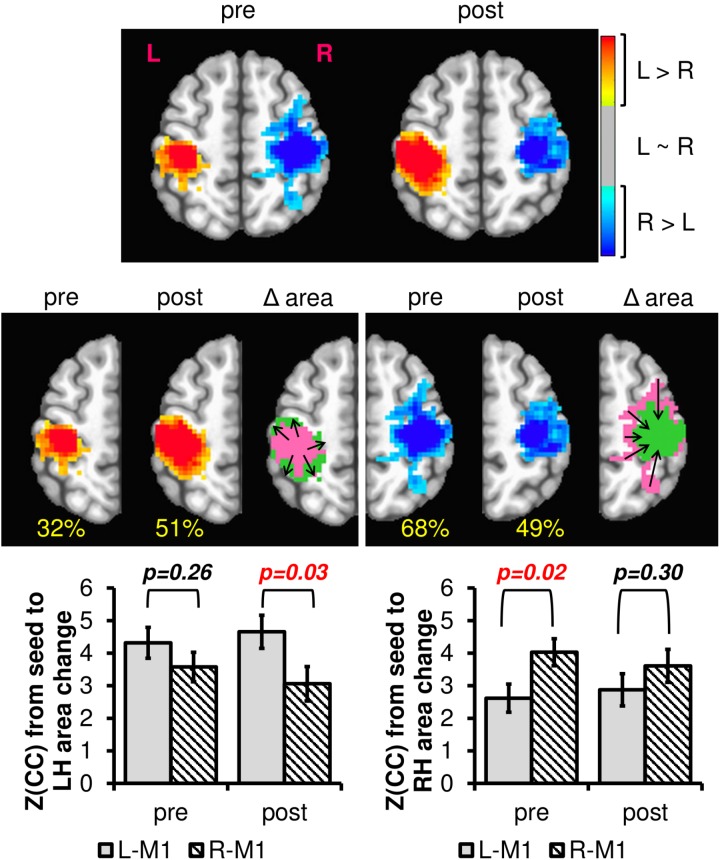
The difference in left and right M1 connectivity (L-R_M1_), as calculated with Equation 4 PRE and POST 12-week exercise intervention. The hot colors represent areas where the left M1 is more connected than the right M1, and the cool colors represent the opposite. The blank areas contain voxels where left and right M1 have similar or no connectivity. The percentage values in yellow denote the proportion of voxels within one hemisphere compared to total of both hemispheres within each session. Notice that from pre to post, the left hemisphere L-R_M1_ area expands (from 32 to 51%), whereas the right hemisphere L-R_M1_ area contracts (68–49%). Plotting the average connectivity strength in the voxels with changing area, R-M1 connectivity to the left hemisphere is reduced after 12-weeks of exercise intervention while the L-M1 connectivity is maintained or slightly increased. L-M1 connectivity increases and R-M1 connectivity decreases in the voxels with changing areas. After a 12-week exercise intervention (AE or BAL), the group average M1 connectivity profile is balanced across hemispheres. Δarea pink = pre area, Δarea green = post area.

Previous research has noted changes in patterns of M1 activity as a function of regular engagement in physical activity (McGregor et al., 2011, 2013; Hassanlouei et al., 2017; Lulic et al., 2017). However, we are unaware of any work to date that has addressed interhemispheric connectivity changes between right and left M1 using rsfMRI. To determine if resting brain networks could detect related changes in interhemispheric connectivity patterns due to exercise intervention, we computed the difference in left to right M1 connectivity separately for PRE and POST across all subjects. As seen in Figure 7, the L-M1 connectivity profile increases due to the 12-week exercise intervention, both in extent (i.e., area) and magnitude, whereas the R-M1 connectivity profile decreases due to the intervention. In effect, the motor network is changed from being more bilateral in PRE to more consolidated to the dominant M1 in POST.

## Discussion

A prominent finding in the current report is that a relatively brief intervention of only 12-weeks was associated with changes in connectivity between the default mode network and sensorimotor networks. Previous studies have employed a longer duration of over 6 months to a year to show similar changes in connectivity (Voss et al., 2010a; Flodin et al., 2017; Rosano et al., 2017; Baniqued et al., 2018). That our aerobic exercise group showed greater connectivity of sensorimotor regions with the default mode network might denote the effectiveness of an interval-based spin exercise intervention in induction of cortical plasticity, as shown in Figure 4. Because there was some overlap in heart rate responses between groups, we collapsed across groups and assessed the relationship between heart rate response (% of time spent in the aerobic training zone) and change in connectivity from pre- to post- intervention. Moreover, across intervention type, time spent in aerobic zone best described not only the imaging data, but also improvements in motor function, as described also in McGregor et al. (2018).

Previous studies of aging-related changes in resting state connectivity have consistently shown that sedentary aging is associated with decreased connectivity of the default mode network (being comprised of the medial frontal cortex, posterior cingulate/retrosplenial areas, and precuneus) (Zheng et al., 2015; Sun et al., 2016). A reduction of connectivity respective of aging, particularly in anterior regions of the DMN has been associated with declines in processing speed and executive function (Damoiseaux et al., 2008). This reduction in connectivity due to age is concerning because alteration of anterior default mode connectivity has been reported in aging-related pathologies such as mild cognitive impairment (Jin et al., 2012), depression (Andreescu et al., 2009) and Alzheimer’s disease (Greicius et al., 2003). Interestingly, recent studies have shown that physical interventions of durations greater than 24 weeks have increased connectivity in the DMN, suggesting that physical activity may reverse aging-related DMN connectivity decreases. In the current study, we found that over both 12-week physical activity interventions, if participants spent more time in the aerobic training zone, DMN connectivity to primary motor cortex increased more. When we then tested the changes in anterior default mode network connectivity respective of change in heart rate throughout the 12-week interventions, we found increased connectivity not only to the motor networks, but also increased connectivity strength within the entire DMN. This finding is worth further consideration.

Studies involving physical activity interventions have shown that sensorimotor networks show greater connectivity in concert with default mode networks, however, the interrelationship between networks is far from clear (Burzynska et al., 2015; Flodin et al., 2017). The default mode network can be functionally split into nodes comprised of the aDMN, as noted above, and the posterior DMN (pDMN). The pDMN is comprised of regions of the precuneus in the lateral portions and retrosplenial cortices in the posterior cingulate areas (Raichle and Raichle, 2001; Damoiseaux et al., 2008). Our results demonstrate when seeded from the primary motor cortex, greater time spent in the aerobic training zone was positively correlated with increased connectivity between both anterior and posterior regions of the DMN (as shown in Figures 4, 5). The functional implications of these increases in connection strength between regions is currently unknown. Though speculative, the increased connectivity between the dominant M1 and pDMN respective of time in aerobic HR zone may indicate an increased state of self-readiness or awareness (Buckner and Carroll, 2007). As such, increased levels of physical activity may serve to enhance the transition of attention to engagement (sometimes considered intention), which has been associated with activity in pDMN, but is lowered as a function of sedentary aging (Andrews-Hanna et al., 2007). Additional research is required to further validate this finding respective of effects on motor behavior.

Interestingly, the most robust correspondence of motor connectivity changes involve the prediction of changes in aDMN based on the amount of time spent in the aerobic zone (% Time in Target_50_ HR Zone). The correlated connectivity changes between aDMN and motor regions (Figures 4, 5) might reflect an increase in preparatory motor control after the interventions. Numerous studies have reported that the aDMN serves in a regulatory capacity in resting state performing activities such as visualization and evaluation (Molnar-Szakacs and Uddin, 2013; Uddin, 2013; Davey et al., 2016). Increased connectivity between aDMN and M1 may be associated with improved motor selectivity prior to transitioning from resting state to task engagement. While additional research into this relationship is clearly required, this change in top-down regulation of motor control may, in part, be responsible for alteration of cortical inhibition in M1 after exercise (Voelcker-Rehage et al., 2010; Maddock et al., 2016; McGregor et al., 2018).

In the current study, we found that after physical activity interventions across participants, the extent of M1 connectivity was increased in dominant M1 and decreased in non-dominant M1 (Figure 7). Notably, decreased influence of non-dominant hemisphere after exercise interventions has been shown to improve motor performance in older adults (McGregor et al., 2011, 2018). Moreover, this reduction in motor cortical network connectivity after exercise may indicate a selective maintenance of networks that promote motor performance. Recently, King et al. (2017) reported that aging-related decreases in motor performance are associated with an increase in internetwork connectivity. The group found that more widespread connectivity may indicate a breakdown of functional segregation of motor networks (King et al., 2017). These data may be analogous to findings in task-based functional MRI that have shown decreased bilateral M1 recruitment during motor hand tasks in highly physically trained older individuals and that this may represent changes in cortical inhibition across hemispheres (Voelcker-Rehage et al., 2010; McGregor et al., 2013). However, the present data require additional investigation to better understand if increases in connectivity after an aerobic intervention represent changes in intracortical inhibition or facilitation respective of changes in motor performance.

In the present study, we found the amount of time spent in aerobic heart rate zone proved to be more sensitive to Pre–Post intervention comparisons in resting state connectivity than VO_2_. This finding is noteworthy for a number of reasons. The first of which is that our sample included primarily highly sedentary individuals many of whom had not engaged in any structured or otherwise physical activity regimen in decades. The YMCA method of VO_2_max estimation requires multiple time points at which to measure heart rate. After three stages of increasing intensity, the reliability of the measurement is quite good for younger adults (ACSM, 2013), but has recently been shown to be less accurate for older adults (Garatachea et al., 2007; Jamnick et al., 2016). Indeed, at study enrollment, participants were not able to achieve a heart rate steady state within each testing segment due to their highly deconditioned nature. This caused some measurement error in the assessment, which may be responsible for the lack of sensitivity of the test with respect to the brain imaging data. However, it should also be noted that some participants even after the spin intervention had difficulty with the YMCA test. Difficulty post-treatment was related to the level of exertion (i.e., Target HR) throughout the intervention and occurred for participants in both the spin and balance interventions. This is consistent with the fact that any intervention has responders and non-responders. The measurement of time spent in aerobic zone across sessions (% Time in Target_50_ HR Zone) largely accounted for the response differences within groups. That is, despite exercise prescription and encouragement to work at a given heart rate level, participants had different responses to the programs. While participants were encouraged to remain below aerobic training zones in the balance and stretching condition, on average, the group spent over 50% of their sessions in an aerobic zone. While this finding on its face may indicate problems with study design and execution, it is worthwhile to note that many non-aerobic interventions show similar results (Voss et al., 2010a; Kleemeyer et al., 2017; Prehn et al., 2017). For example, Prehn et al. (2017), enrolled obese older adults into either an aerobic (cycling) or non-aerobic (stretching) intervention arm. Curiously, their stretching group improved on VO_2_peak testing while their aerobic group evidenced no change (Prehn et al., 2017). Yet the exercise group showed cortical changes in connectivity in similar networks where change occurred in the present study. Additionally, Flodin et al. (2017) had participants engage in either cardiovascular exercises (running/cycling/cross-training) or repetitive strength training over 6 months. Both groups showed significant improvement in estimated VO_2_max. Importantly, there were no significant differences in any cortical connectivity changes when comparing across groups. However, when the authors used VO_2_ change as a predictor across groups, post-intervention increases in DMN and M1S1 network connectivity were revealed (Flodin et al., 2017). In addition, Burzynska et al. (2015) reported that level of physical activity as measured by accelerometry predicted changes in network connectivity (Burzynska et al., 2015).

This commentary is not to say that aerobic capacity or cardiovascular improvement is not a driving mechanism of change in exercise studies. For example, Voss et al. (2016) clearly showed that cardiovascular function accounted for more variability than physical activity level in their rsfMRI data. These relate directly to the current work, as the group found that high fitness older adults had much higher connectivity within the anterior DMN and dorsal attention networks (Voss et al., 2016). Moreover, work from our own lab has repeatedly shown that cardiovascular fitness (measured by estimates of VO_2_ change) likely is the neuroprotective factor in slowing aging related declines in both language and motor control (McGregor et al., 2011, 2013, 2018; Zlatar et al., 2013; Nocera et al., 2017). The more salient point is that studies need to be extremely careful with respect to design of the intervention when working with sedentary older adults. Physical activity interventions in this population are notoriously difficult to manage given the multifactorial nature of physical activity within and beyond a specified exercise regimen. The use of physical activity tracking outside of the study environment with respect to heart rate and total movement (accelerometry) is now a vital component to any exercise intervention. The recent increase in availability and decrease in cost of commercially available technologies to track these measures offers unprecedented opportunities (and potentially additional challenges) in related work and should be incorporated into future projects. Further, the biomolecular correlates of change after physical activity interventions needs to be closely tracked, as multiple studies have shown the impact of serum-levels of BDNF on cortical function (Erickson et al., 2012; Huang et al., 2014; Vaughan et al., 2014).

## Conclusion

The current study found that 12-week physical activity interventions alter connectivity between the default mode network and cortical motor networks when accounting for time spent in aerobic HR zone. Interestingly, prior to interventions the connectivity of primary motor areas was disproportionately distributed between hemispheres with the non-dominant hemisphere showing a greater extent of resting state connectivity. After interventions, the connectivity profile became less bilateral with larger extent of connectivity in M1 in the dominant hemisphere. These data may indicate a functional change in motor cortical resting state connectivity as a result of increased physical activity over a 12-week intervention.

## Author Contributions

KMc, JN, and BC design of experiment. KMc, JN, and KMa implementation of intervention and management of intervention. KH, KMc, LK, VK, JO, and KMa data processing. KMc, LK, VK, JN, and KG data analysis. KMc, LK, and JN manuscript writing.

## Conflict of Interest Statement

The authors declare that the research was conducted in the absence of any commercial or financial relationships that could be construed as a potential conflict of interest.
